# Potential for natural evaporation as a reliable renewable energy resource

**DOI:** 10.1038/s41467-017-00581-w

**Published:** 2017-09-26

**Authors:** Ahmet-Hamdi Cavusoglu, Xi Chen, Pierre Gentine, Ozgur Sahin

**Affiliations:** 1 Department of Chemical Engineering, Columbia University, New York, New York 10027 USA; 2 Department of Biological Sciences, Columbia University, New York, New York 10027 USA; 3 Department of Earth and Environmental Engineering, Columbia University, New York, New York 10027 USA; 4 Department of Physics, Columbia University, New York, New York 10027 USA; 5 Present Address: Advanced Science Research Center (ASRC) at the Graduate Center of The City University of New York, New York, New York 10031 USA; 6 Department of Chemical Engineering, The City College of New York, New York, New York 10031 USA

## Abstract

About 50% of the solar energy absorbed at the Earth’s surface drives evaporation, fueling the water cycle that affects various renewable energy resources, such as wind and hydropower. Recent advances demonstrate our nascent ability to convert evaporation energy into work, yet there is little understanding about the potential of this resource. Here we study the energy available from natural evaporation to predict the potential of this ubiquitous resource. We find that natural evaporation from open water surfaces could provide power densities comparable to current wind and solar technologies while cutting evaporative water losses by nearly half. We estimate up to 325 GW of power is potentially available in the United States. Strikingly, water’s large heat capacity is sufficient to control power output by storing excess energy when demand is low, thus reducing intermittency and improving reliability. Our findings motivate the improvement of materials and devices that convert energy from evaporation.

## Abstract (web summary)

The evaporation of water represents an alternative source of renewable energy. Building on previous models of evaporation, Cavusoglu et al. show that the power available from this natural resource is comparable to wind and solar power, yet it does not suffer as much from varying weather conditions.

## Introduction

**Fig. 1:**
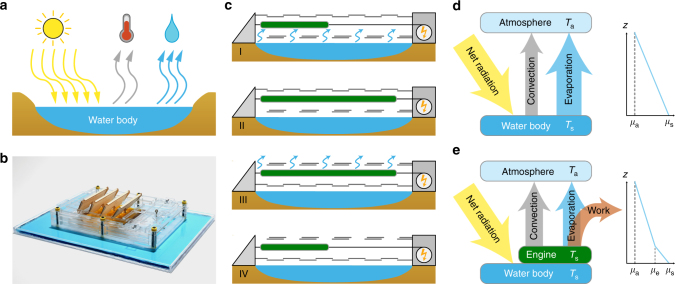
The surface energy balance in the absence and presence of an evaporation-driven engine. **a** The net radiative energy into a water body is balanced by convection and evaporation. **b** An example of an evaporation-driven engine, incorporating water-responsive materials, placed at the water surface can harness energy from evaporation^12^. **c** Such an engine harnesses energy from evaporation through a 4 stage cycle: (I) With the upper shutters (*gray jagged line*) closed, the water-responsive material (*green block*) swells, absorbing water vapor at the high chemical potential *μ*_s_. (II) At maximum absorption, the upper shutters open as the bottom shutters close. (III) With the upper shutter open, the water-responsive material shrinks, releasing water that evaporates away into the atmosphere at a lower chemical potential *μ*_e_. (IV) At maximum desorption, the upper shutters close as the bottom shutters open, restarting the engine cycle. **d** The flows between the water body and the atmosphere occur along a thermal gradient between *T*_s_ and *T*_a_ for convection and along a chemical gradient between *μ*_s_ to *μ*_a_ for evaporation. **e** The new energy balance can be illustrated between net incoming radiation, convection, evaporation, and work extracted between *μ*_s_ and *μ*_e_

Evaporation, with an average global energy flux of about 80 W m^−2^, is a powerful process in nature^1–3^ that affects ecosystems, water resources, weather, and climate^4–7^. Recent advances in water responsive materials^8–11^ and devices^12–15^ demonstrate the ability to convert energy from evaporation into work. These materials perform work through a cycle of absorbing and rejecting water via evaporation. These water-responsive materials can be incorporated into evaporation-driven engines that harness energy when placed above a body of evaporating water (Fig. 1a–c). With improvements in energy conversion efficiency, such devices could become an avenue to harvest energy via natural evaporation from water reservoirs. However, little is known about the potential of natural evaporation as a renewable energy source—specifically, the power availability, intermittency, and the impact on water resources.

The evaporation rate *E* is governed by the surface energy balance between net radiation and heat losses due to turbulent convection and evaporation (Fig. 1d). Combining this energy balance with equations of heat and mass transfer can predict *E* over a saturated water surface from meteorological data (i.e., net solar radiation, relative humidity, air temperature, and wind speed)^16^. This model has been adapted to understand changes in *E* over varying surfaces, such as plants^17^ and soil^18, 19^.

In this work, we estimate the power available from natural evaporation from open bodies of freshwater, such as lakes and water reservoirs, by modeling the effects of an evaporation-driven engine on the energy balance and coupled heat and mass transport. We then study the power potential of natural evaporation from open water surfaces in the United States–along with the potential impact of such evaporation driven engines on water resources and energy reliability. We find that natural evaporation could provide power at areal densities up to 10 W m^−2^ (triple that of modern wind power) along with evaporative water losses being cut by nearly half. When restricted to existing lakes and reservoirs larger than 0.1 km^2^ in the contiguous United States (excluding the Great Lakes), we estimate the total power available to be up to 325 GW, which is over 69% of the US electrical energy generation rate in 2015. Finally, we investigate the possibility to control power output from an evaporation driven engine by using water’s heat capacity to store and release energy. Strikingly, we find that storing energy thermally in the water below an evaporation driven engine could substantially reduce intermittency by varying power supply to match power demand.

## Results

### A relationship between power and evaporation rate

An evaporation-driven engine placed just above the water surface is powered by absorbing water at a high chemical potential, *μ*_s_, and releasing it at a lower chemical potential, *μ*_e_, to the atmosphere, *μ*_a_ (*μ*_s_ > *μ*_e_ > *μ*_a_; Fig. 1e). For a reversible and isothermal engine, the power output depends on *E* and the work done per mole of evaporating water *w* = *μ*_s_–*μ*_e_. However, one cannot simply multiply existing *E* data by *w*, as the energy conversion process alters the evaporation rate. Therefore, predicting the power available from natural evaporation requires a relationship between *w* and *E*.

*E* is affected by *w* in two ways. First, the chemical potential drop *w* across the engine results in a reduction in water vapor pressure across the engine, which reduces the mass transport. In the case of an ideal gas^20^, *w* is − *RT*_s_ ln(*α*), where *R* is the molar gas constant, *T*_s_ is the temperature of the surface, and *α* is the ratio of the vapor pressures above and below the engine. Note that the air immediately above the water surface is saturated with water vapor, therefore the ratio *α* is also the relative humidity at the top of the engine (in dimensionless units 0.00–1.00)^21^. We can rewrite *α* as follows:

1 $$\alpha \left( w \right) = {e^{\frac{{ - w}}{{R{T_s}}}}}$$

Because the evaporation rate depends on the vapor pressure deficit between the engine surface and the atmosphere, an increase in *w* causes a reduction in evaporation rate. Second, the total energy required to evaporate water and extract energy from an evaporation-driven engine is the sum of the latent heat *L* and the work energy *w*. We define the ratio of this total energy to the unperturbed case as *β*:

2 $$\beta \left( w \right) = \frac{{L + w}}{L}$$

Here, *L* is the molar latent heat of vaporization of water in J/mol. Thus, *β* represents the energy penalty for evaporating water through an evaporation-driven engine versus the case with no engine. Consequently, *w* affects the energy balance between net radiation and heat loss due to convection and evaporation, because some portion of the energy from net radiation is now removed from the system as work.

Using parameters *α* and *β*, it is possible to derive a model that predicts the evaporation rate and power generated from it. Note that *w* can be dynamically adjusted during operation by varying the resistance of the load so that the water responsive material in the engine must exert a larger force on the load. Thus, it is possible to control *α* and *β*. At steady-state, the net radiation leaves the engine surface via convection, evaporation (i.e., latent heat), and power generation. The convective heat flux is proportional to the temperature difference between the engine surface and the atmosphere, whereas the latent heat flux is proportional to the difference in vapor pressures between the engine surface and the atmosphere. The magnitudes of these two energy fluxes also depend on the transport characteristics of the air, which is primarily determined by turbulence and wind speed. Using these relationships, we derived an equation that relates the latent heat flux, *F*, to *α* and *β* (Methods):

3 $$F = \frac{{\alpha \Delta }}{{\alpha \beta \Delta + \gamma }}\left( {I + \frac{\gamma }{{\alpha \Delta }}f\left( u \right)\left( {\alpha - {\rm RH}} \right){p_a}} \right)$$

Here, *f*(*u*) is the convective mass transport coefficient of water vapor as a function of wind speed *u*, *I* is the net radiation, *Δ* is the slope of the saturation vapor pressure versus. temperature curve, *γ* is the psychrometric constant, RH is the relative humidity of the air, and *p*_a_ is the saturated vapor pressure of water at the air temperature. Equation (3) shows that evaporation occurs even when the net radiation is zero, as long as the relative humidity of the air is less than *α*. Under this condition, the remaining term in the parenthesis can be viewed as the drying power of the sub-saturated atmosphere.

Once *F* is calculated, the evaporation rate *E* can be obtained from the relationship *F* = *ELρM*_v_, where *ρ* and *M*_v_, are the respective liquid density and molecular weight of water. Finally, the areal power density *W* is given by *W* = *Fw*/*L*.

### Power generation and evaporative losses vary with weather conditions

**Fig. 2:**
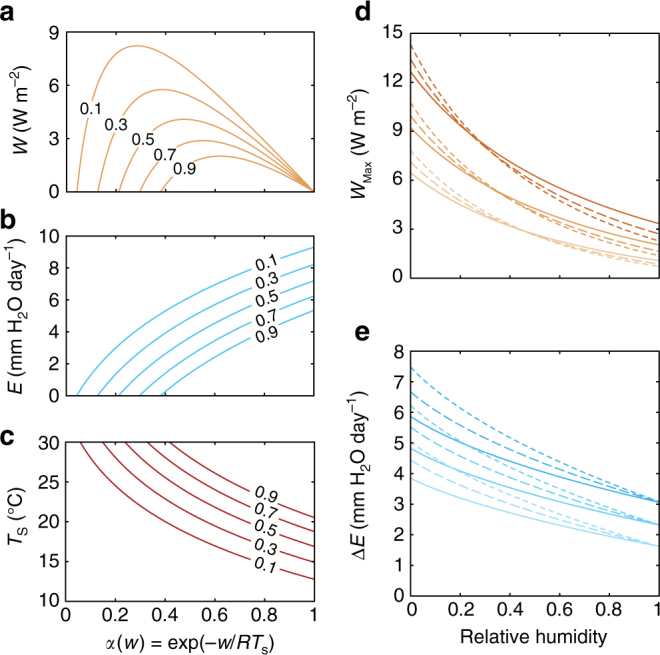
Steady-state power generation and effects on evaporative losses. **a** Energy fluxes, **b** evaporation rates, and **c** surface temperatures are calculated as a function of *α*(*w*,*T*_s_) for weather conditions of 200 W m^−2^ *I*, 16 °C *T*_a_, 101.3 kPa *P*, and 2.7 m s^−1^ (6 mph) *u* at 5 values of RH (mild conditions). **d** Maximum energy flux and **e** water saved from evaporation as a function of RH at cool (*pale*, 12 °C, 150 W m^−2^), mild (*neutral*, 16 °C, 200 W m^−2^), and warm (*dark*, 20 °C, 250 W m^−2^) weather conditions and three wind speeds: 1.8 (4 mph, *solid*), 2.7 (6 mph, *dashed*), and 3.6 m s^−1^ (8 mph, *dotted*)

Figures 2a, b illustrates predictions for *W* and *E* as a function of *α*(*w*, *T*_s_) for a range of RH values at conditions representative of typical mild weather conditions. As *α* is lowered from unity (*w = *0), the surface temperature rises while *E* gradually falls (Fig. 2b, c). This gradual increase in surface temperature results in a proportional increase in convective heat losses *C*. Evaporation ultimately stops at a certain *α* value, at which point heat is released mostly as convective heat *C*. Importantly, *W* peaks at an optimal *α* value (i.e., an optimal *w* that maximizes the power density for given weather conditions). Interestingly, *E* at optimal power density is approximately half the open water *E* (*α* = 1) under the same weather conditions (Supplementary Fig. 1).

To better understand which weather variables most influence the optimal power density, we plot the optimal power densities and corresponding evaporation rate reductions as a function of relative humidity for a range of weather conditions (Fig. 2d, e). Interestingly, we find that the optimal power density varies weakly with wind speed, and increases strongly with decreasing atmospheric relative humidity. We also find that the potential water savings increases with increasing wind speed and decreasing relative humidity. The results suggest power densities of up to 15 W m^−2^ and parallel evaporation rate reductions up to 7.5 mm H_2_O per day at some of the warmest and driest conditions. Note that these conditions vary over time and geography. For example, the distribution of daily relative humidity values at Daggett-Barstow, California shows that the days where the relative humidity falls below 40% occurs about 65% of the time (Supplementary Fig. 2). Therefore, one has to take into account the variability of weather conditions to determine the average power available.

**Fig. 3:**
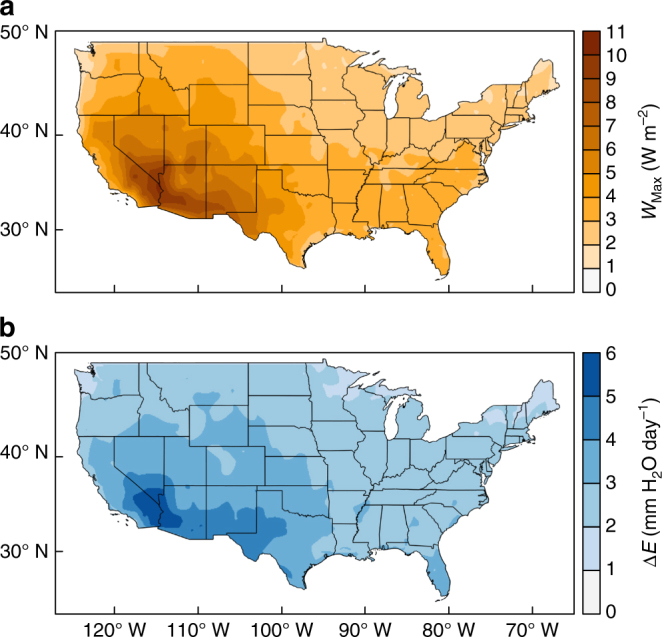
Maps of power generation from natural evaporation and water savings. **a** Maximum power density available and **b** total decrease in evaporation rate due to power harvesting potentially available from open water surfaces across the contiguous United States of America. Maps calculated using the data^22^ across 934 weather stations to calculate *W*_Max_ and corresponding Δ*E* at each location from eq. (3) with natural neighbor interpolation and linear extrapolation to generate a 5′ resolution map

Using regional meteorological data^22^, our model can now provide insight into the distribution of power densities available. By calculating maximum daily *W* and averaging it across an entire year, we generate a 5′ resolution map of power density and parallel water savings across the contiguous USA (Fig. 3 and Supplementary Fig. 3). These maps suggest average annual power densities and corresponding water savings up to 10.49 W m^−2^ and 5.9 mm H_2_O per day, respectively. These maximums are located at Needles Airport in California, only 11 km from Goose Lake and 47 km from Lake Havasu. This result is particularly striking since the locations of peak power potential and water savings occur simultaneously in the US Southwest, a region that frequently suffers from water scarcity. As a point of reference, the current mean total area power densities for current US wind and photovoltaic installations are 2.90 and 8.06 W m^−2^, respectively^23, 24^.

The data in Fig. 3 allow us to predict the total power and water savings potentially available from lakes and reservoirs in the US via a database of open water bodies^25^. By identifying the location and surface area of each open water body, we predict the potential annual mean power output and corresponding water savings available at each water body if it was covered entirely with an ideal evaporation driven engine. Our analysis reveals that 325 GW (2.85 million MWh per year) is potentially available by covering lakes and reservoirs larger than 0.1 km^2^ across the contiguous US (excluding the Great Lakes). Additionally, an additional 96.4 billion cubic meters of water could be recovered each year due to lower evaporation rates. Our results shown in Table 1 indicate that potential power available exceeds demand in 15 of 47 US states studied^26^, and saves more freshwater than consumed in 7 of those 15 US states^27^. The summary results of all US states studied can be found in Supplementary Table 1.

**Table 1:** US States where the potential power available due to evaporation from open water surface area exceeds the net energy generation rate

US State	Open water surface area (km^2^)	Potential power available (MW)	Potential water savings (10^6^ m^3^ per year)	Net energy-generation rate (MW)	Freshwater withdrawals (10^6^ m^3^ per year)
Utah	8393.0	47,200.53	10,540.70	4788.71	5711.02
California	4844.8	27,550.54	6376.01	22,454.78	43,048.52
Minnesota	8996.0	19,251.52	6651.15	6504.54	5279.30
Louisiana	4413.7	14,353.23	4704.11	12,307.35	11,804.42
Nevada	1710.4	12,292.26	2586.21	4457.40	3614.10
Oklahoma	2729.3	9831.92	3159.98	8691.28	2454.63
Oregon	2382.9	8994.33	2332.57	6605.77	9312.79
Montana	2854.4	8628.27	2615.48	3345.02	10,546.27
Maine	4029.0	8357.80	2845.18	1340.33	564.93
South Dakota	3030.5	7617.27	2762.17	1099.66	864.67
Idaho	1816.9	6896.89	1795.02	1788.48	23,806.20
North Dakota	2831.9	6833.77	2425.13	4241.62	1566.52
Wyoming	1420.4	6004.67	1543.46	5589.79	6414.11
New Mexico	598.6	3734.85	874.37	3733.04	4366.53
Vermont	1246.7	2775.62	1018.56	226.26	595.77

### Potential effects of feedback between the engine and the atmosphere

Our estimates of steady state evaporation rates and power do not currently consider potential changes in atmospheric conditions due to the reduction in evaporation rates. This can be viewed as a feedback interaction between the engine and the atmosphere. Such feedback mechanisms can be critical to distributed renewable energy systems. For example, atmospheric feedback imposes limits to the maximum power generation of wind turbines^28, 29^. Therefore, it is important to consider potential feedback effects in our model.

One potential feedback pathway is caused by the changes on the atmosphere due to covering lakes and reservoirs with evaporation-driven engines. The evaporation-driven engine reduces the evaporation rate while increasing the rate of convective heat loss (due to higher surface temperatures). This shift of energy from evaporation to convection mimics the conditions seen when moist soils become dry, where higher convective heat fluxes warm the air due to reduced water availability for evaporation. Previous studies^30–36^ show that as previously moist soil become drier, the atmosphere becomes more arid, consistently shifting toward higher air temperatures and lower relative humidities^37, 38^. These changes contribute toward a reduction in cloud cover^39, 40^ (i.e., an increase in net radiation). Individually, these changes would increase the potential for evaporation that could result in power densities greater than those for fixed weather conditions, as seen in eq. (3).

Another feedback pathway is to expand the total available area for evaporation driven engines. This could be due to artificially creating new reservoirs. This would have the opposite effect; with more open water surfaces made available, more evaporation would occur, leading to reduced air temperature and increased humidity. Such feedback has been shown in studies involving large-scale changes in land-use (e.g., urbanization, irrigation)^41, 42^. This would result in power densities lower than those for fixed weather conditions.

However, the magnitude of these feedback pathways is likely to be small for the daily mean temperature and would primarily modify temperature extremes^43^. Globally, any changes that could occur in the atmosphere is small since ocean evaporation dominates total global evaporation and the resulting temperature and humidity responses^44, 45^. Locally, feedback effects will also be small if the dimensions covered by an engine are below 500 km (ref. ^46^). This is due to the important role of horizontal heat and moisture transport in the atmosphere that couples neighboring regions. Therefore, we are neglecting potential feedback effects, as they would not drastically affect our estimates.

### Control of power output under varying weather conditions

While the model described by eq. (3) allows estimating power density and its dependence on meteorological variables, the ability to predict variability of power from evaporation at short timescales is limited due to the approximation that the net heat storage in the body of water is negligible. Evaluating this variability is crucial to understand the potential of evaporation as a renewable energy source since many renewable energy technologies suffer from intermittent availability.

To explore the variability of power from evaporation, we incorporate heat storage in the body of water below an evaporation driven engine into the energy balance among net radiation, evaporation, convection, and power generation. To approximate the heat storage, we assume a simple mixed-layer water body with density *ρ*, specific heat capacity *c*_w_, and mixed-layer depth *d* (i.e., the epilimnion; typically, at least 5 m deep for lakes larger than 1 km^2^)^47, 48^. The energy balance is then given by (Methods, Supplementary Fig. 4):

4 $$\rho d{c_{\rm{w}}}\frac{{\partial {T_{\rm{s}}}}}{{\partial t}} = I - \beta F - C$$

Here, the rate of heat storage is balanced by incoming net radiation (*I*) and outgoing convective heat losses (*C*) and the sum of latent heat flux (*F*) and power output (*W*). Note that *βF* = *F* + *W*. Thus, eq. (4) allows us to predict the water temperature *T*_s_, the latent heat flux *F*, and the power density *W* as a function of the chemical potential drop *w* and changing weather conditions over time. Importantly, *w* can be independently controlled. This feature might allow us to control power generation, potentially mitigating the effect of changing weather conditions.

**Fig. 4:**
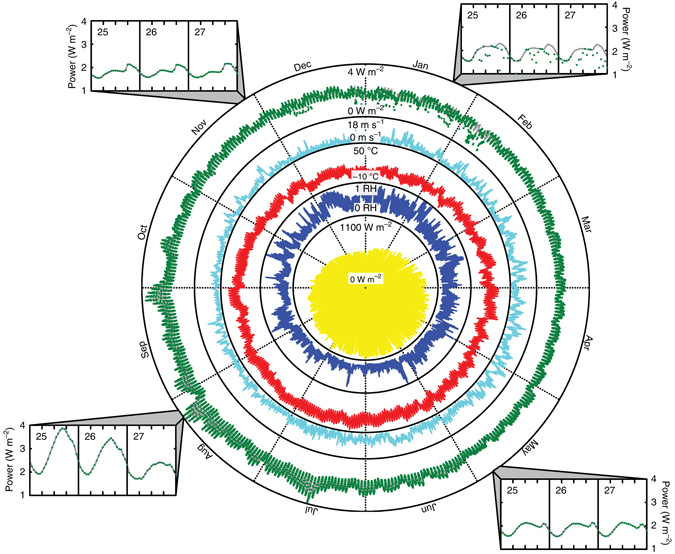
Matching variable demand by controlling power output via heat storage. Results for the final year of a simulation run for South-East Central California from Daggett-Barstow, California. From inside-out: Hourly (1) *I* (*yellow*, W m^−2^), (2) RH (*blue*, %), (3) *T*_a_ (*red*, °C), (4) *u* (*cyan*, m s^−1^), (5) *W*_PD_ (*gray*, W m^−2^) and predicted *W*_O_ (*green dots*, W m^−2^). Clockwise from the top-right are 3-day samples of hourly *W*_PD_ (*gray*, W m^−2^) and predicted *W*_O_ (*green dots*, W m^−2^) for January, May, August, and November. Despite the variability of power demand and weather, power generation matches demand 95% of the time. Meteorological data^22^ and power demand data^49^ are from publically available databases. Annual data are evenly divided by hourly data

To demonstrate this, we develop a control system that adjusts *w* to match a power demand target over time (Supplementary Fig. 5). We set the system’s power demand to that of three major U.S. energy markets in 2010 (South-East Central California^49^, North Central Texas^50^, and New York City^51^) along with their respective varying typical weather conditions^22^. Because the power output of an evaporation driven engine scales with area, we are interested in relative variations in power demand over time rather than absolute values. Thus, we normalize each power demand curve to a target annual mean power density. Figure 4 illustrates the results of a simulation year in California with a target annual mean power demand of 2 W m^−2^. The results show that power generation matches demand 95% of the time, exhibiting some shortages on winter days where net radiation is low and relative humidity is higher. Supplementary Fig. 6 illustrates results for Texas (93% match) and New York (67% match).

**Fig. 5:**
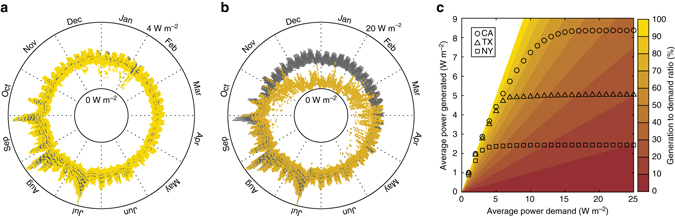
The relationship between reliability and average power output. The demand (*gray line*) and generation (*dots*) for (**a**) 2 W m^−2^ and (**b**) 10 W m^−2^ annual average demand targets for the final simulation year in California. In **a**, generation matches demand 95% of the time with 99% annual generation to demand ratio. In **b**, generation matches demand 48% of the time with 71% annual generation to demand ratio. **c** Predicted average power generation as a function of target power demand for California (*circles*), Texas (*triangles*), and New York (*squares*) test locations. The overlaid contour map is the resulting generation to demand ratios at each power demand condition for that specific average power generation. These simulations predict that the maximal generation is 2.4, 5.1, and 8.4 W m^−2^ for the respective New York, Texas, and California test locations

As the annual mean power demand increases, the frequency of power shortages increases despite an increase in the mean power generation. Figures 5a, b illustrates this aspect by comparing the 2 W m^−2^ case to a 10 W m^−2^ case in California. As this comparison shows, the 10 W m^−2^ case suffers from more power shortages during cooler months and is only able to match demand 48% of the time. However, some power generation still occurs during these cooler months resulting in the system’s annual generation-to-demand ratio to climb above 80%.

To better understand the relationship between generation and demand, we repeat these calculations for a range of mean power demands. Figure 5c plots mean generation versus mean demand at each test location along with a generation-to-demand ratio heat map (see also Supplementary Fig. 7 for water savings versus mean demand). As demand increases, the system eventually saturates and provides no more additional generation. These simulations predict a maximum generation of 2.4, 5.1 and 8.4 W m^−2^ for the respective New York, Texas, and California test locations. Compared to the map in Fig. 3a, the control system delivers at least 85% of the power generation predicted by eq. (3) (2.8, 5.3, and 8.4 W m^−2^ for the respective New York, Texas, and California locations). Importantly, as the imposed generation target is reduced, the reliability of the system to match power demand increases.

## Discussion

By developing a model of how an evaporation-driven engine perturbs the evaporation rate, this work provides the first predictions on how these energy harvesters could optimally perform in the natural environment. Although these evaporation energy harvesters are in the early stages of development, recent advances water-responsive materials^8–11^ and devices^12–14^ suggest several pathways toward achieving the predicted performance levels. With advances in energy conversion performance, these materials and devices could potentially contribute toward solving energy and water related challenges.

Figure 3 illustrates the broad availability of energy and water savings by covering a water reservoir with evaporation driven engines. These maps show that the regions of highest potential power generation and water savings are located where water is scarce. This is striking because water and energy are typically seen as competing challenges while harnessing natural evaporation could address both challenges at once. As an example, we consider the possibility of converting the E.V. Spence Reservoir in Texas (31.93°N 100.57°W) into an evaporation power plant. If this reservoir (38.0 km^2^ surface area in 2004)^25^ is completely covered by an evaporation driven engine, it would generate an average annual power output of 178 MW. This is 62 MW (53%) greater than the nearby Sweetwater Phase IV Wind Farm^23^. Moreover, the E.V. Spence Reservoir, which has been drastically impacted by a recent multi-year drought^52^, could benefit from the potential water savings as a result of energy harvesting.

It is important to note that using evaporation driven materials and devices on lakes or reservoirs could affect freshwater resources^53^ (e.g., altering the water withdrawal rate, gas exchanges, water quality, and recreational use). These consequences would impose additional design and planning constraints on such systems that could reduce the area available for energy harvesting. However, the potential area available for open water energy harvesting is substantial—lakes and reservoirs cover at least 95,000 km^2^ (excluding the Great Lakes) of the contiguous United States^25^—and are found across a geographically diverse range of locations^54^. Some of these regions suffer from periods of water stress and scarcity^52^, which might favor implementation of these energy harvesting systems due to the reduction of evaporative losses.

Finally, a key challenge for current renewable energy resources is intermittency: wind turbines^55^ and solar photovoltaic panels^56^ produce power only when the wind and the sun are available. Since the supply of power must match demand on a real-time basis to maintain a stable electric grid, energy storage is a critical component of stable renewable energy systems to mitigate intermittency^57^. The results in Figs. 4, 5 show that the natural thermal energy storage capability of water is potentially sufficient to match realistic power demand variability. This is a dramatic result for a renewable energy source that depends on variable environmental conditions. Thus, natural evaporation could provide a way to address the intermittency problem of renewable energy. Therefore, these findings suggest that natural evaporation could potentially be a widely available source of low-intermittency renewable energy.

## Methods

### Derivation of equation (3)

For steady-state evaporation, the incoming energy from net radiation leaves the water surface via convection and evaporation (i.e., latent heat). The energy flux due to convection is proportional to the temperature difference between the surface and the atmosphere, whereas the energy flux due to evaporation is proportional to the difference in vapor pressures between the surface and the atmosphere. We can eliminate the need to have surface temperature data by combining these fluxes with the energy balance^16^.

By introducing the power output due to water evaporating through an evaporation-driven engine placed above a water surface, this new energy balance is

5 $$I = F + W + C$$

This system is now described by the energy balance between net radiation energy *I* (solar plus longwave) into a body of water against the energy losses through evaporative latent heat flux *F*, power per unit area *W*, and convective heat flux *C*.

The latent heat flux *F* is proportional to the vapor pressure deficit between the engine and the atmosphere and a mass transfer coefficient

6 $$F = f\left( u \right)\left( {\alpha {p_{\rm{s}}} - {\rm RH}{p_{\rm{a}}}} \right)$$

Here, *f*(*u*) is the mass transport coefficient and *αp*_s_ – RH *p*_a_ is the vapor pressure deficit between the engine surface (*αp*_s_) and the sub-saturated atmosphere (RH *p*_a_). The Clausius–Clapeyron relation describes the relationship between the change in saturation vapor pressure of water *p* and the change in temperature *T*, which yields the following form when the molar latent heat of vaporization *L* is assumed to be constant

7 $$p = \exp \left( {18.371 - \frac{{5132}}{T}} \right)$$

Here, *p* is in kPa and *T* is in K. A more accurate relationship is given by the Antoine equation. However, differences are negligible for the temperature ranges involved in this analysis.

The convective heat flux is proportional to the temperature difference between the engine and the atmosphere and a heat transfer coefficient

8 $$C = \gamma f\left( u \right)\left( {{T_{\rm{s}}} - {T_{\rm{a}}}} \right)$$

Here, the psychrometric constant *γ* (units kPa K^−1^) represents the ratio between the heat capacity of moist air to the latent heat of water, and combined with *f*(*u*) represents the heat transport coefficient^58^. The similarity in the relationships between *F* and *C* is because the fundamental mechanisms of heat and mass transport are essentially the same for water vapor in the air (i.e., the Reynolds analogy). For this work, we have used an empirical transport coefficient:^59^

9 $$f\left( u \right) = 74.43(1 + 0.536u)$$

This equation calculates the value of *f*(*u*) in W m^−2^ kPa^−1^ when *u* is given in m s^−1^ at 2 m height.

We can couple *F*, *C*, and *W* together to simplify the right-hand side of the energy balance in eq. (5) as a function of the latent heat flux *F*. By our definition of *β* in eq. (2) as the ratio of the total engine energy to latent heat, *F* *+* *W* *=* *βF*. Likewise, because of the similarity in the relationships between the latent heat flux *F* and the convective heat flux *C*, we can express *C* as a ratio to *F*. This simple ratio is known as the Bowen ratio^58^

10 $$B = \gamma \frac{{{T_{\rm{s}}} - {T_{\rm{a}}}}}{{\alpha {p_{\rm{s}}} - {\rm {RH}}{p_{\rm{a}}}}}$$

We can now rewrite the energy balance as *I* *=* *F*(*β* + *B*). However, this equation still requires currently unknown surface temperature data to solve. To reduce the need for surface temperature data, we use the Clausius–Clapeyron relation *Δ*, which is the slope of the vapor pressure versus temperature curve

11 $$\Delta \equiv \frac{\partial }{{\partial T}}p\left( T \right) = \frac{L}{{R{T^2}}}p\left( T \right) \approx \frac{{{p_s} - {p_a}}}{{{T_s} - {T_a}}}$$

We can now estimate the temperature difference between the surface and the atmosphere by using (*p*_s_–*p*_a_)/*Δ*, thus eliminating the need to know the surface temperature to predict the thermal gradient in eq. (10). We can now rewrite the Bowen ratio from eq. (10) as

12 $$B = \frac{\gamma }{\Delta }\frac{{{p_{\rm{s}}} - {p_{\rm{a}}}}}{{\alpha {p_{\rm{s}}} - {\rm RH}{p_{\rm{a}}}}}$$

However, we still need a relationship to eliminate our dependence on surface vapor pressure data. To address this challenge, we introduce the latent heat flux of the atmosphere

13 $${F_a} = f\left( u \right)\left( {\alpha {p_{\rm{a}}} - {\rm RH}{p_{\rm{a}}}} \right)$$

Here, the surface vapor pressure *p*_s_ for *F* in eq. (6) has been replaced with the saturated vapor pressure of the atmosphere *p*_a_. Thus, *F*_a_ represents the drying power of the sub-saturated atmosphere if the surface was at the same temperature as the air. Therefore, the ratio of *F*_a_ to *F* is

14 $$\frac{{{F_{\rm{a}}}}}{F} = 1 - \alpha \frac{{{p_{\rm{s}}} - {p_{\rm{a}}}}}{{\alpha {p_{\rm{s}}} - {\rm RH}{p_{\rm{a}}}}}$$

We can use *F*_a_/*F* in eq. (12) to estimate the ratio between the saturation vapor pressure deficit due to temperature differences (*p*_s_–*p*_a_) and the true vapor pressure deficit between the engine and the sub-saturated atmosphere (*αp*_s_–RH*p*_a_), thus eliminating the need to know the surface temperature to predict the vapor pressure gradient.

By re-writing the Bowen ratio from eq. (12) with this new information, we get

15 $$B = \frac{\gamma }{{\alpha \Delta }}\left( {1 - \frac{{{F_{\rm{a}}}}}{F}} \right)$$

We use eq. (13) and (15) to solve *I* *=* *F*(*β* + *B*) and get the expression for *F* in eq. (3)

$$F = \frac{{\alpha \Delta }}{{\alpha \beta \Delta + \gamma }}\left( {I + \frac{\gamma }{{\alpha \Delta }}f\left( u \right)\left( {\alpha - {\rm RH}} \right){p_{\rm{a}}}} \right)$$

There are two important caveats to this model. First, we have not completely eliminated the need to know the surface temperature for this model, since it is used to set *α* in eq. (1). Second, we need to choose a temperature to evaluate *Δ* in eq. (11) to estimate the ratio of the saturation vapor pressure deficit (*p*_s_–*p*_a_) to the thermal gradient (*T*_s_–*T*_a_). We can address both issues through an iterative approach.

For the first iteration, we approximate both *α* and *Δ* at the air temperature. After determining *F*, we re-approximate the surface temperature *T*_s_ by using the aerodynamic equation for the convective heat flux *C* in eq. (6) and the Bowen ratio shown in eq. (15)

16 $${T_{\rm{s}}} = {T_{\rm{a}}} + \frac{{F - {F_{\rm{a}}}}}{{\alpha \Delta f\left( u \right)}}$$

This is an improved estimate of the surface temperature for *α*. Next, we calculate the mean temperature between the air and surface, *T*_m_ = (*T*_s_ + *T*_a_)/2, for solving *Δ* in eq. (13). This provides a better estimate of the ratio between the saturation vapor pressure deficit (*p*_s_ – *p*_a_) and the thermal gradient (*T*_s_ – *T*_a_). With these improvements, we can iterate through eqs. (3), (16) until the surface temperature converges toward a solution.

### Generation of geographical maps

For our steady state model, we generate daily mean *I*, *T*_a_, RH, *u*, and *P* values at each TMY3 station in the contiguous USA (934 total stations) and calculate the maximum power output and corresponding water savings for that day. This calculation is repeated for 365 days in the dataset, and the annual average power output and corresponding water savings is calculated across these 365 samples (Supplementary Fig. 3). The annual mean at each location is then used to develop the geographical maps in Fig. 3 by natural-neighbor interpolation.

### Calculation of total power and water savings possible

For our steady-state model, we identify the location and size of each contiguous lake and reservoir in the Global Lakes and Wetlands Database^25^ found within the contiguous United States. We then interpolate between our data from Fig. 3 to calculate the total power generation and corresponding annual water savings possible for that location if the entire water body was covered with an evaporation driven engine. Additionally, the distance to the nearest TMY3 weather station and the US Air Force code of that station is stored for each station. The results of this calculation are used to develop the summary statistics shown in Supplementary Table 1. Data from the Energy Information Administration^26^ and US Geological Survey^27^ are used to determine the respective net energy generation rate and freshwater consumption in each state.

### Derivation of equation (4)

Our non-steady state (dynamic) model is exactly described by the energy balance between net radiation energy *I* (solar plus longwave) into a body of water against the energy losses through evaporative latent heat flux *F*, power density *W*, convective heat flux *C*, horizontal conduction *G*, and heating of the water *S* from the water body:

17 $$I = F + C + W + S + G$$

The horizontal conduction *G* represents the heat transfer due to the difference in temperatures between the water and the soil of the shore. Over the longer time scales of the steady-state analysis, *G* is estimated to be negligible. To continue disregarding this heat transfer in the shorter time scales being explored, we assume that the sides and the bottom of the water body are insulated. The remaining energy flows out of the body of water is power, evaporation, and convection. The evaporative heat flux *F* is defined in eq. (6), the convective heat flux *C* is defined in eq. (8), and the power density *W* is defined by *W* = *F w*/*L*.

The final remaining item in the energy balance is the heat storage term *S*. We describe *S* with a lumped capacitance model. In this model, the energy storage capability of the body of water is proportional to the heat capacity of water and the change in temperature over time

18 $$S = \rho d{c_{\rm{w}}}\frac{{\partial {T_{\rm{s}}}}}{{\partial t}}$$

Here, *ρ* is the density of water, *d* is the epilimnion depth of water (the warmest, near isothermal, upper layer of a body of water), *c*_w_ is the heat capacity of water, and *∂T*_s_ */∂t* is the rate of change in water temperature over time due to heat storage/loss.

By substituting our new expressions for *F, C*, *W*, and *S*, we rearrange the energy balance of eq. (16) to produce eq. (4)

4 $$\rho d{c_{\rm {w}}}\frac{{\partial {T_{\rm{s}}}}}{{\partial t}} = I - \beta F - C$$

We confirm that the non-steady-state energy balance defined by eq. (4) does converge toward the steady state energy balance of eq. (3) (Supplementary Fig. 4a–c). Due to the storage term *S*, the water surface temperature depends on the past history of the energy balance, thus exhibiting a memory effect. The time needed for this system to forget the past is called the relaxation time, which is strongly dependent on the depth of water *d* (Supplementary Fig. 4d), with some additional dependence on the wind speed *u* and the work load *w*.

### Generation of simulation data for power demand matching

TMY3 data from stations 723815 (Daggett-Barstow Airport), 722650 (Midland International Airport), and 725020 (Newark International Airport) are used to provide hourly typical meteorological data for our three respective test markets in California, Texas, and New York. To simulate the varying power demand for each respective test location, each hourly regional power load data set is normalized by the respective mean power load for 2010 and then scaled by a pre-factor to gauge the potential power density of this power system. Hourly data is linearly interpolated to generate data at one-second intervals for calculations, with data sampling at one-hour intervals.

### Derivation of controller parameters

To control the power delivery of the model system (Supplementary Fig. 5a), a combined feedback and feedforward controller with saturation limits is designed (Supplementary Fig. 5b). By looking at Fig. 2, it is evident that operating on the high *w* (low *α*) side of the *W* curve would lead to lower evaporation rates (therefore greater water savings) and higher thermal energy storage. To achieve this, a feedforward model is designed where the *α* required for zero evaporation, *α*_0_, is defined at any moment by knowing the current *T*_s_, *T*_a_, and RH. This is defined as the ratio between the sub-saturated vapor pressure in the air and the saturation vapor pressure at the water surface:

19 $${\alpha _{\rm{0}}} = \frac{{{p_{\rm{d}}}}}{{{p_{\rm{s}}}}} = \frac{{{\rm RH}{p_{\rm{a}}}}}{{{p_{\rm{s}}}}}$$

This solution can be used to determine the *w*_0_ required for zero evaporation:

20 $${w_{\rm{0}}} = L\left( {\frac{{{T_{\rm{s}}}}}{{{T_{\rm{a}}}}} - 1} \right) - {\rm R}{{\rm T}_{\rm{s}}}\log \left( {\rm RH} \right)$$

For convenience, we use *α* in our implemented ideal Proportional-Integral (PI) controller scheme. The controller gain is tuned to the inverse slope of the *W*(*α*) curve at *α*_0_

21 $$K = \frac{{\partial \alpha }}{{\partial W}}{|_{{\alpha _0}}} = - \frac{L}{{f\left( u \right){p_s}R{T_{\rm{s}}}\log {\alpha _{\rm{0}}}}}$$

However, to reduce the computational time, an estimated gain of 0.0015 m^2^ W^−1^ is used in this work. The integral time is tuned to the time step of the simulation, one second in this work. A saturation range of 0.0–0.2 is applied to the PI feedback controller to prevent controller overshoot due to the non-linearity of the system. A final saturation control range of 0.0001–1.0 is applied to the sum of the feedforward and feedback controllers to prevent illogical controller values. The clamping anti-windup method provided by MATLAB-Simulink is used to prevent PI controller overshoot due to saturation.

The non-linear characteristics demonstrated by the power density versus surface vapor pressure curve in Fig. 2a can be adequately linearized near the zero-evaporation and zero work condition (low *α*). However, at higher power density levels, there is a risk of the controller going ‘over the hill’ and leading to a catastrophic failure of the PI control scheme. To avoid this, we use saturation controls to design a relatively safe controller at the cost of losing out on the maximum power potential of the system. It may be of interest to investigate alternative control methods to further improve the output of the proposed power plant system.

### Code availability

The code used for this work is accessible in figshare^60^.

### Data availability

The revised TMY3 data^22^ is accessible at http://rredc.nrel.gov/solar/old_data/nsrdb/1991-2005/tmy3. 2010 real-time power demand data was downloaded from the California Independent System Operator (CAISO) OASIS database^49^, the Electric Reliability Council of Texas (ERCOT) Hourly Load Data Archive^50^, and the New York Independent System Operator (NYISO) Custom Report database generator^51^. The data that support the findings of this study are available in figshare^60^.

## Electronic supplementary material

Supplementary Information
