# Supplementary material for: Potential for natural evaporation as a reliable renewable energy resource
Source: Nat Commun. 2017 Sep 26;8:617. doi: 10.1038/s41467-017-00581-w (PMC5615039; doi:10.1038/s41467-017-00581-w)
Supplement: Supplementary file 1 — Supplementary Information [file 41467_2017_581_MOESM1_ESM.pdf]

Description of Supplementary Files

File Name: Supplementary Information
Description: Supplementary Figures and Supplementary Table

US State	Open Water Surface Area (km^2)	Potential Power Available (MW)	Potential Water Savings (10^6 m^3 / year)	Net Energy Generation Rate (MW)	Freshwater Withdrawals (10^6 m^3 / year)
Utah	8,393.0	47,200.53	10,540.70	4,788.71	5,711.02
California	4,844.8	27,550.54	6,376.01	22,454.78	43,048.52
Texas	5,835.2	21,557.70	7,105.39	51,350.04	31,330.44
Minnesota	8,996.0	19,251.52	6,651.15	6,504.54	5,279.30
Florida	5,778.5	18,516.16	6,555.36	27,101.90	8,572.70
Louisiana	4,413.7	14,353.23	4,704.11	12,307.35	11,804.42
Nevada	1,710.4	12,292.26	2,586.21	4,457.40	3,614.10
Oklahoma	2,729.3	9,831.92	3,159.98	8,691.28	2,454.63
Oregon	2,382.9	8,994.33	2,332.57	6,605.77	9,312.79
Montana	2,854.4	8,628.27	2,615.48	3,345.02	10,546.27
Maine	4,029.0	8,357.80	2,845.18	1,340.33	564.93
South Dakota	3,030.5	7,617.27	2,762.17	1,099.66	864.67
Tennessee	2,435.0	7,471.78	2,301.29	8,586.15	10,644.95
Idaho	1,816.9	6,896.89	1,795.02	1,788.48	23,806.20
North Dakota	2,831.9	6,833.77	2,425.13	4,241.62	1,566.52
North Carolina	2,259.9	6,759.28	2,301.30	14,656.22	15,295.17
Alabama	2,096.0	6,743.71	2,080.63	17,406.10	13,815.00
Wisconsin	2,873.7	6,460.54	2,212.82	7,575.36	8,511.02
Wyoming	1,420.4	6,004.67	1,543.46	5,589.79	6,414.11
Arkansas	1,693.5	5,725.01	1,742.70	6,342.40	15,665.22
Georgia	1,657.8	5,430.77	1,726.02	14,705.24	6,130.40
Washington	1,887.3	5,280.05	1,616.29	12,475.74	6,808.82
New York	2,459.3	5,230.50	1,871.86	15,825.08	7,918.95
Missouri	1,602.2	5,153.40	1,609.49	9,547.95	11,853.76
South Carolina	1,503.9	4,889.18	1,595.62	11,019.66	9,374.46
Michigan	2,000.4	4,317.96	1,541.75	12,900.46	14,925.13
New Mexico	598.6	3,734.85	874.37	3,733.04	4,366.53
Virginia	1,154.0	3,428.69	1,137.83	9,636.03	6,130.40
Arizona	403.0	3,407.94	710.25	12,915.76	8,412.35
Colorado	634.2	2,917.65	735.66	5,980.94	15,171.83
Kansas	898.8	2,795.83	976.10	5,197.16	5,538.33
Vermont	1,246.7	2,775.62	1,018.56	226.26	595.77
Illinois	972.4	2,620.09	887.48	22,140.64	18,008.83
Kentucky	850.9	2,503.63	768.84	9,536.95	5,982.39
Mississippi	703.0	2,420.52	753.45	7,392.45	5,328.64
Nebraska	635.1	2,081.10	677.34	4,552.90	11,113.67
New Hampshire	586.2	1,434.13	457.15	2,284.92	506.96
Iowa	534.7	1,284.57	463.81	6,467.91	4,243.18
Ohio	506.6	1,164.58	428.32	13,914.77	13,074.91
Massachusetts	500.3	1,117.73	407.11	3,662.78	1,467.84
Pennsylvania	456.4	1,104.55	375.42	24,494.55	11,249.35
Indiana	421.9	1,066.98	371.42	11,874.35	11,952.44
Maryland	277.7	771.23	264.53	4,151.32	2,035.25
New Jersey	258.4	705.17	244.93	8,516.99	2,676.66
Connecticut	157.1	353.80	125.81	4,277.47	1,128.64
West Virginia	111.7	297.77	91.46	8,252.88	4,884.59
Rhode Island	43.1	98.34	36.31	792.13	186.26
Grand Total	95,486.7	325,433.81	96,403.85	462,709.22	419,888.32

Supplementary Table 1 | Summary statistics of open water surface area, potential power generation from natural evaporation, and concurrent water savings along with net energy generation rate and freshwater consumption data, by US State

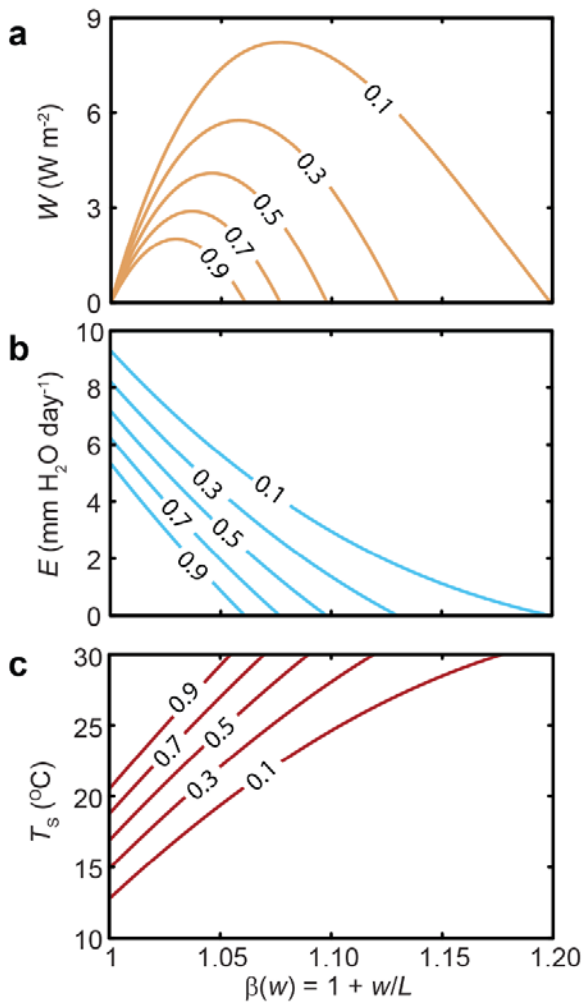

Supplementary Figure 1 | Steady-state power generation and effects on evaporative losses as a function of β a, Energy fluxes **b,** evaporation rates, and **c,** surface temperatures are calculated as a function of $\beta(w)$ for weather conditions of 200 W m^{-2} *I*, 16 ^{o}C T_a, 101.3 kPa *P*, and 2.7 m s^{-1} (6 mph) *u* at 5 values of *RH* (mild conditions). $\beta(w)$ depends on the load *w* (work done per mole of water evaporated).

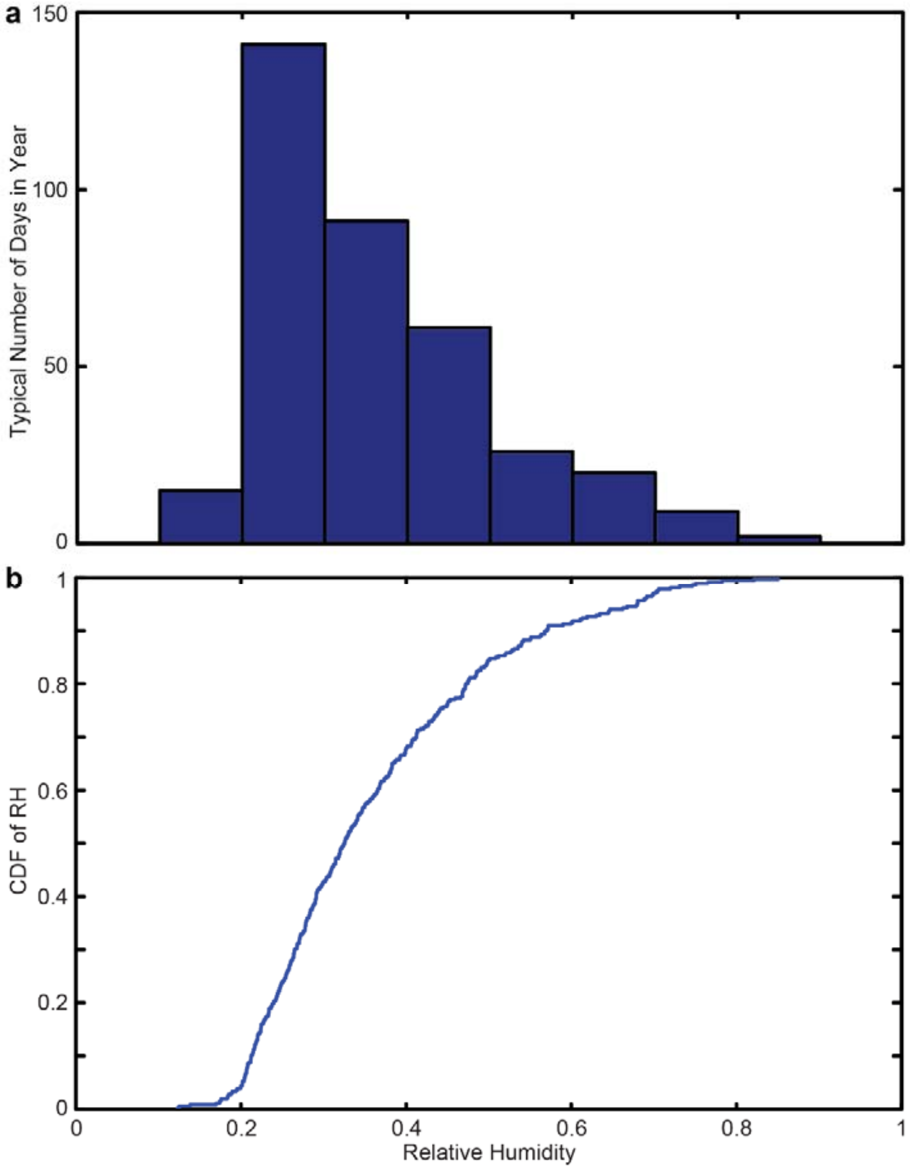

Supplementary Figure 2 | Distribution of typical daily relative humidity in Daggett-Barstow, CA a, Histogram and **b,** empirical cumulative distribution function of typical daily relative humidity in Daggett-Barstow, California. The mean relative humidity is 36.2%, observed over 365 daily mean *RH* values from TMY3 data for the Daggett Barstow, California TMY3 data set.

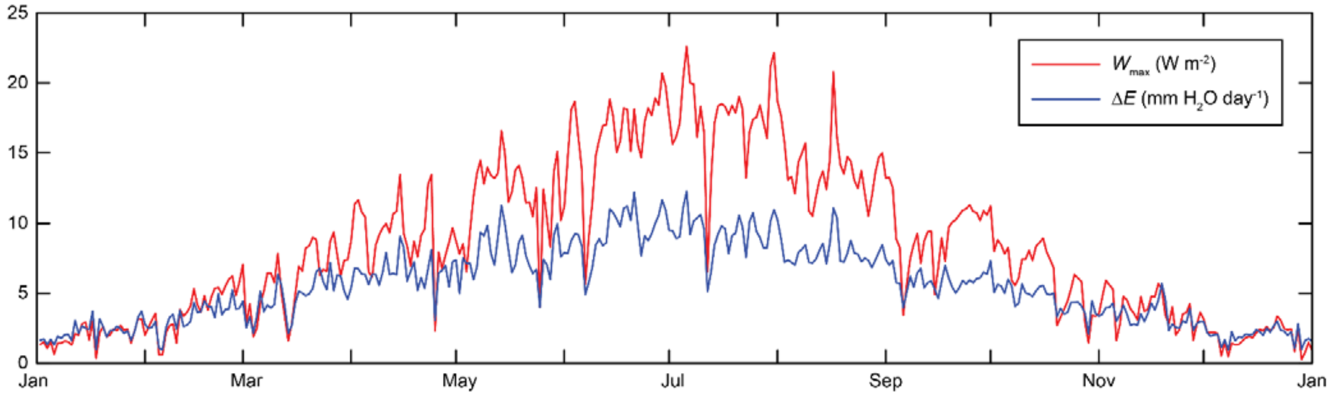

Supplementary Figure 3 | Daily prediction of peak power generation and corresponding water savings in Daggett-Barstow, California Daily maximum power output (W m^{-2}, blue) and corresponding water savings (mmH_2O day^{-1}, red) predicted by Equation 3 for Daggett Barstow, California. Input data is generated by using daily mean I, T_a, P, u, and RH values from TMY3 data for the Daggett-Barstow, California TMY3 data set.

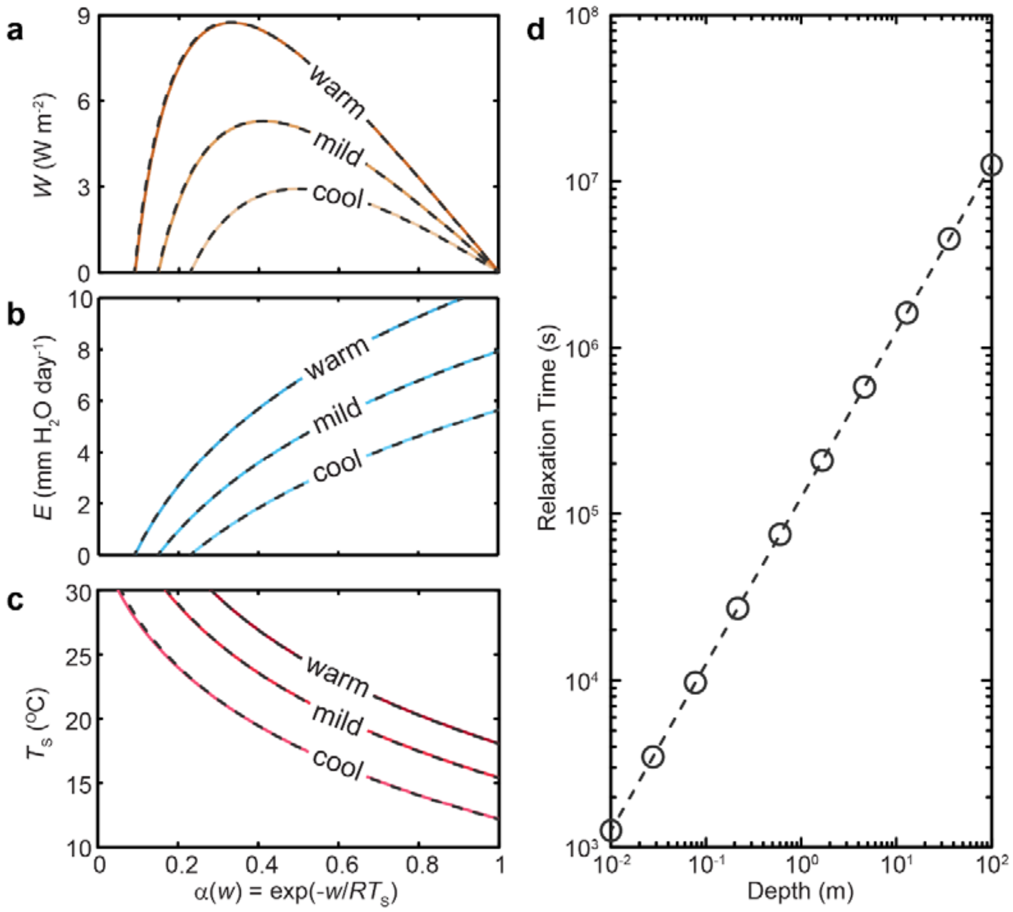

Supplementary Figure 4 | Heat balance power generation model converges toward steady-state prediction and estimates of energy storage a, Energy fluxes, **b,** evaporation rates, and **c,** surface temperatures of three selected conditions. Steady state results are solid lines, the final non-steady state results are dashed lines. The non-steady state results converge toward the steady state predictions after one simulation year. Results are calculated for cool (pale, 12 ^{o}C, 45% RH, 150 W m^{-2}), mild (neutral, 16 ^{o}C, 35% RH, 200 W m^{-2}), and warm (dark, 20 ^{o}C, 25% RH, 250 W m^{-2}) weather conditions at 2.7 m s^{-1} (6 mph). The dotted lines show the convergence of a non-steady state model initialized at a surface temperature of 288 K with an isothermal depth of 5 m. The relaxation times of the surface temperature is plotted as a function of isothermal water depth **d,** for ten selected depths (circles) and interpolated (dashed line) at mild (16 ^{o}C, 35% RH, 200 W m^{-2}) conditions.

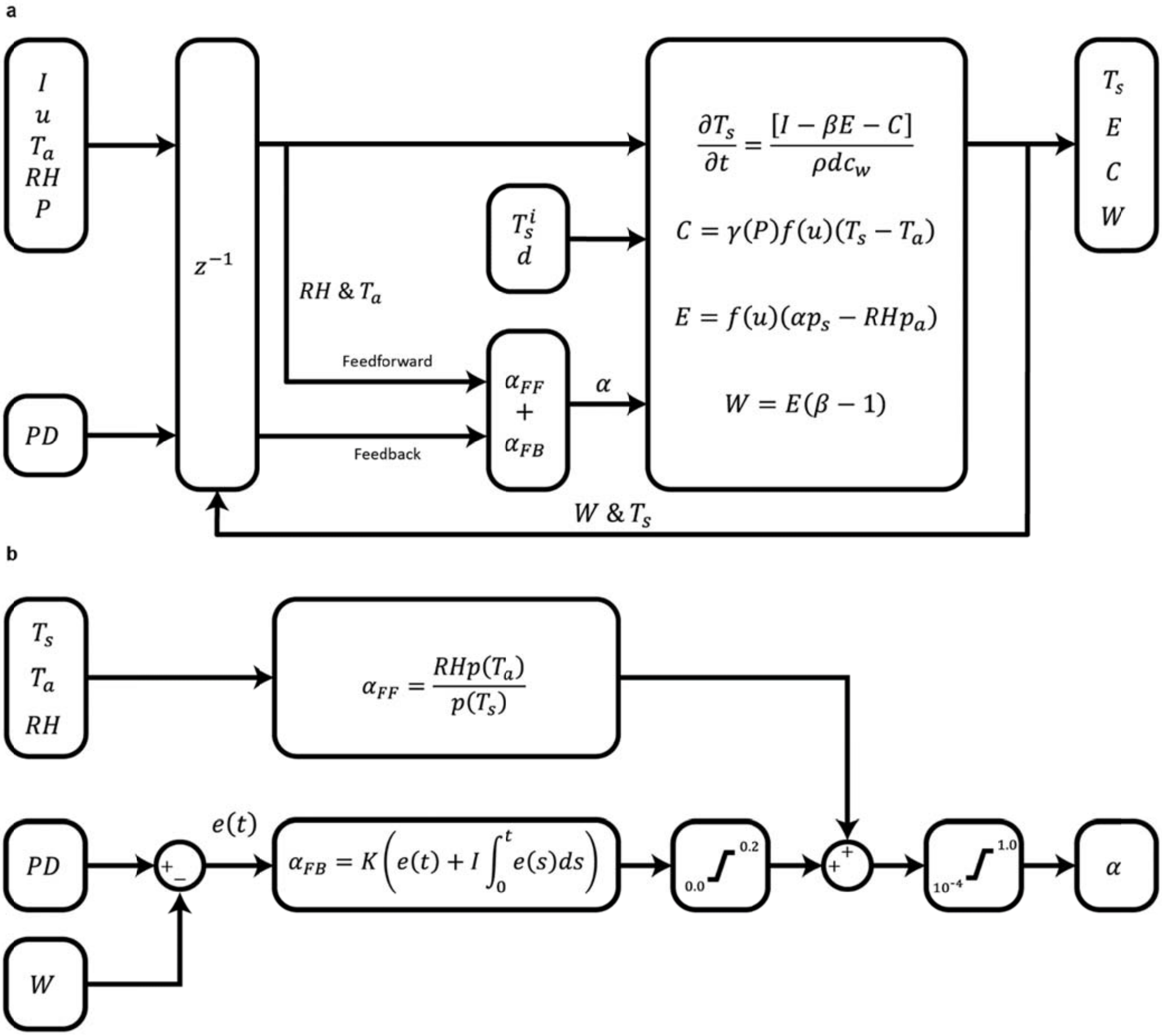

Supplementary Figure 5 | Simulation model of a controlled evaporation driven engine a, Process model for an evaporation energy harvesting power plant and **b,** the control model for simulating the reliability of such a power plant.

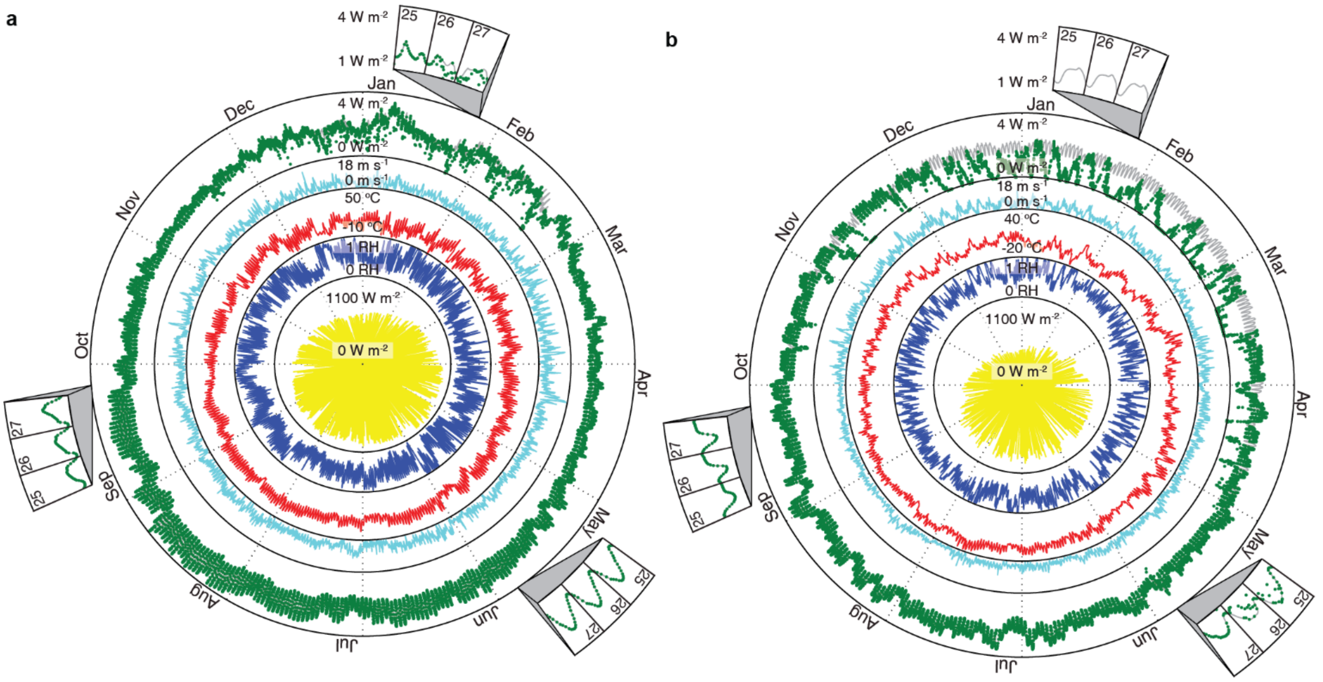

Supplementary Figure 6 | Matching variable demand by controlling power output via heat storage Results for the final year of a simulation run for **a,** North Central Texas from Midland, Texas and **b,** Greater New York City from Newark, New Jersey. From inside-out: Hourly 1) I (yellow, W m^{-2}), 2) RH (blue, %), 3) T_a (red, ^{o}C), 4) u (cyan, m s^{-1}), 5) W_{PD} (gray, W m^{-2}) and predicted W_O (green dots, W m^{-2}), and 6) three 3-day zoomed in samples of hourly W_{PD} (gray, W m^{-2}) and predicted W_O (green dots, W m^{-2}). The results show that power generation matches demand **a,** 93% and **b,** 67% of the time. Meteorological data is from the TMY3 data set. The power demand data is from the **a,** ERCOT database or **b,** NYISO database. Annual data is evenly divided by hourly data.

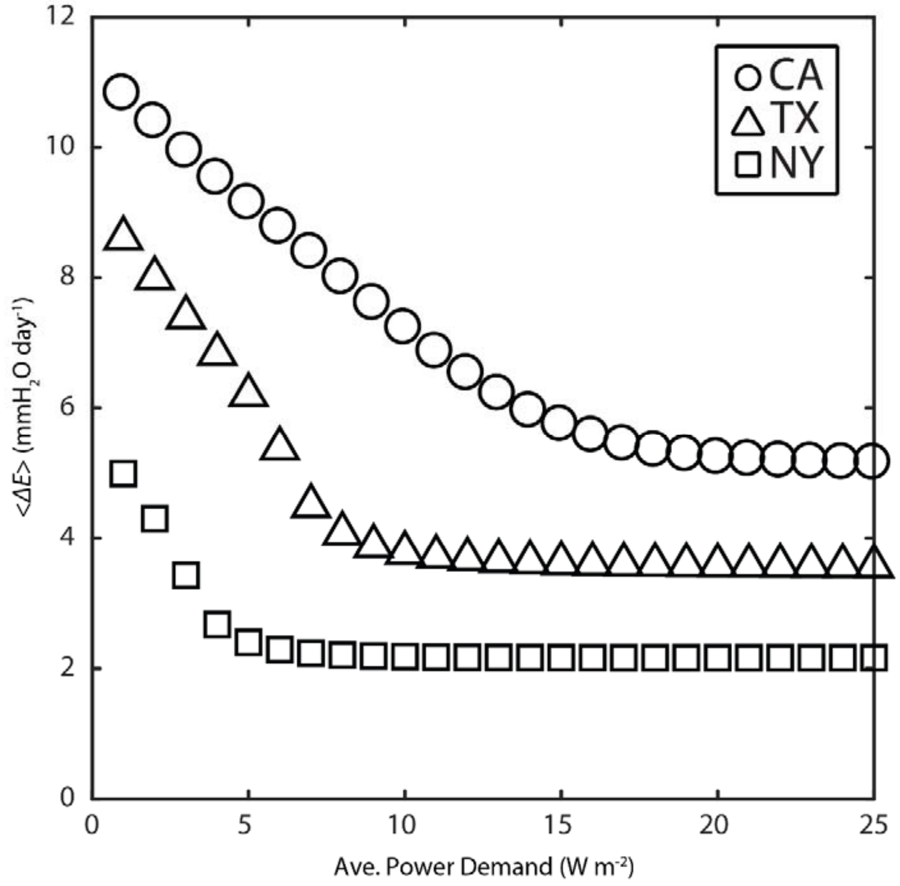

Supplementary Figure 7 | The relationship between water savings and power demand Predicted water savings as a function of target power demand for California (circles), Texas (triangles), and New York (squares) test locations. These simulations predict that the minimum water savings is 2.2, 3.6, and 5.1 mmH_2O day^{-1} for the respective New York, Texas, and California test locations.
